# *Perilla frutescens* Britton: A Comprehensive Study on Flavor/Taste and Chemical Properties During the Roasting Process

**DOI:** 10.3390/molecules24071374

**Published:** 2019-04-08

**Authors:** Jookyeong Lee, Da-Som Kim, Jinju Cho, Seong Jun Hong, Jeong Hoon Pan, Jae Kyeom Kim, Eui-Cheol Shin

**Affiliations:** 1 Department of Food Science, Gyeongnam National University of Science and Technology, Jinju 52725, Korea; tracylee0911@gmail.com (J.L.); kim94dasom@naver.com (D.-S.K.); aacho7@hanmail.net (J.C.); 01028287383a@gmail.com (S.J.H.); 2 School of Human Environmental Sciences, University of Arkansas, Fayetteville, AR 72701, USA; jhpan@uark.edu (J.H.P.); jkk003@uark.edu (J.K.K.)

**Keywords:** *Perilla frutescens* Britton, roasting, taste, electronic-tongue, flavor, sniffing test, GC-olfactometry, chemical properties

## Abstract

This study investigated changes of volatile compounds, sniffing test-assisted sensory properties, taste associated-constituent and free amino acid compositions, taste description by electronic-tongue, and chemical characteristics in *Perilla frutescens* Britton *var. acuta* Kudo after roasting at 150 °C for 0–8 min. A total of 142 volatile compounds were identified, among which methyl benzoate and limonene were predominant, regardless of roasting time, and these were also detected as the major compounds in the sniffing test by GC-olfactometry. For constituent amino acids analyzed by the acid hydrolysis method using hydrochloric acid (HCl), the concentration of glutamic acid, aspartic acid, and leucine showed an increase pattern with increased roasting time, which results in umami taste, sour taste, and bitter taste, respectively. For free amino acids, valine and hydroxylysine eliciting bitter and bitter and sweet tastes, respectively, also tend to increase by roasting. The pattern of amino acid concentration by roasting was readily matched to the taste description by electronic-tongue but that of sweetness and sourness by electronic-tongue did not coincide with the amino acid composition. For the chemical properties, total phenolic content, antioxidative capacity, and browning intensity tend to increase with roasting but decreased by 8 min. The results of this study provide fundamental information on perilla in both the food industry and cooking environment for the sake of increasing the utilization of perilla as a food source and ingredient.

## 1. Introduction

*Perilla frutescens* is an herbaceous plant that belongs to the Lamiaceae family and grown in Asian countries including Korea, China, Japan, Vietnam, and India [1,2]. It was first cultivated and established as a traditional medicinal plant in China a long time ago [1,3]. *Perilla frutescens* was introduced to Korea in ancient times and has been widely cultivated as a major crop since then [4]. There are various cultivars: *P. frutescens var. frutescens* is consumed as an oil source and fresh vegetable, *P. frutescens var. japonica* as an oilseed, and *P. frutescens var. acuta* and *P. frutescens var. crispa* mainly as fresh vegetables, food ingredients, and medicinal herbs [1,2,3,5]. *P. frutescens var. acuta* and *P. frutescens var. crispa*, are differentiated by the anthocyanin content accumulated in their leaves, whereby they display red-purple and green-purple leaves, and thereby being called red perilla and green perilla, respectively [5].

The literature highlights various biological effects of *Perilla frutescens*. Jun et al., investigated phenolic antioxidant capacity of *P. frutescens var. acuta* leaves [6] and Zhou et al., reported that two phenolic compounds in cold-pressed *P. frutescens var. arguta* seed flour, namely rosmarinic acid and rosmarinic acid-3-O-glucoside, had superior antioxidant activities [7]. In addition to the antioxidative capacity, Jeon et al., identified luteolin in perilla leaves using HPLC and found that luteolin had anti-inflammatory activity against cytokines and antipruritic activity in mice [8]. Further, Kim et al., evaluated the antibacterial activity against *Staphylococcus aureus* of *P. frutescens var. acuta* leaves [9]. Kim et al., discovered that pepsin-hydrolyzed perilla leaf extracts had anti-neuronal damage activity on H_2_O_2_-induced DNA [10]. Lastly, He et al., isolated seven anthocyanins in *P. frutescens var. acuta* and reported anticancer activity against Hela cells [1].

Whereas much research has focused on the physiological and biological benefits of perilla as aforementioned [1,6,7,8,9,10], only a few studies have examined the changes of flavor and taste characteristics and chemical properties after a cooking process such as roasting. Two different studies applied response surface methodology (RSM) to optimize the roasting conditions of perilla leaves as a tea and found that roasting each at 210–220 °C for 10–20 min and 180 °C for 20 min yielded the best quality perilla leaf tea [10,11]. These studies focused on the optimization of roasting conditions but did not examine the alterations of the taste and flavor and of chemical properties over the roasting time. Further, Nam et al., tried to optimize steaming time and onion contents to make perilla pickles [12], and Moon et al., added perilla leaf powder to pork sausages in an investigation of its impact on sausage quality and consumer preference [13]. These two studies introduced perilla as a food ingredient and food additive but, again, did not analyze volatile compounds and the most relevant chemical parameters from a food chemistry perspective.

Therefore, this study aimed to investigate comprehensive changes in *P. frutescens* Britton *var. acuta* Kudo during roasting at 150 °C for various time points (0–8 min); 1) volatile compounds and sniffing test assisted sensory properties to examine flavor changes upon roasting; 2) taste related- constituent and free amino acid compositions and taste description by electronic tongue (E-tongue) to access taste changes by roasting; and 3) total phenolic content (TPC), antioxidative capacity, and browning intensity to describe chemical changes by roasting, in an effort to widen the application of perilla as food ingredients. The results of this study would provide informative data in food industrial applications and serve as a cornerstone in recognizing the potential of perilla as great food sources and food ingredients.

## 2. Results and Discussion

### 2.1. Votile Compounds and Sensory Desciption by GC-Olfactometry

#### 2.1.1. Volatile Compounds Analyzed by GC-MS

Volatile compounds present in *P. frutescens* Britton during the roasting process were collected with SPME and analyzed by GC-MS. The results of changes in the volatile compounds are shown in Table 1. A total of 142 volatile compounds were found in *P. frutescens* Britton *var. acuta* Kudo, among which hydrocarbons (110) accounted for the majority, followed by alcohols (16), miscellaneous compounds (5), aldehydes (5), heterocyclic compounds (3), ethers (2), and ketones (1). Of those volatile compounds, methyl benzoate and limonene were identified as predominant compounds in terms of relative concentration. Methyl benzoate was initially present in high concentration (88.08 μg/100 g) and then it increased to 203.10 μg/100 g at 4 min. It finally showed a substantial decrease after 8 min of roasting (21.31 μg/100 g). Methyl benzoate is an aromatic ester compound eliciting pleasant odors associated with herbal and fruity odors [14,15]. Recent studies demonstrated that methyl benzoate was identified as a major volatile compound in fermented green oranges [15], olives [16], and teas [17]. Similarly, limonene started with a relatively high concentration (54.66 μg/100 g), which increased to 137.24 μg/100 g at 4 min, and then decreased to 60.92 μg/100 g at 8 min. Limonene is an aliphatic terpene usually derived from oils in the peels of citrus fruits invoking citrus-like fruity odors [18]. Researchers described that limonene was one of the key volatile compounds in encapsulated orange oil [19], Chinese wolfberry [20], and Persian lime [21]. In this study, although 8-min roasting seems long enough to decompose both methyl benzoate and limonene, they remained at relatively high contents during the roasting process as they are known to be thermally stable compounds with high boiling points (198 °C [22] and 175–177 °C [22], respectively). Besides those main compounds, there were low quantities of linalool, α-pinene, 2-β-pinene, azulene, dodecane, tetradecane, and caryophyllene which showed varying patterns over time. Dodecane and tetradecane showed good heat resistance, maintaining similar contents during the roasting, with a range of 1.79–2.44 μg/100 g and 1.84–3.15 μg/100 g, respectively. All of these volatiles showed the highest concentration at 4 min except for dodecane, which had the highest at 6 min. Ahmed et al., reported that perilla aldehydes, perilla ketones, limonene, shisofurane, and farnesene were the core compounds among 65 volatile compounds identified in the essential oil of *P. frutescens* (L). Britt [23]. Another study by Seo and Baek found 33 volatile compounds using GC-MS in the leaf of *P. frutescens* Britton with four different extraction methods and identified perilla ketone, (*Z*)-3-hexenol, and 1-octen-3-ol as key compounds [24]. The results of the mentioned studies were slightly discrepant with what we observed in this study, presumably because of the differences in varieties (e.g., Britt vs. Britton) and sample preparation (e.g., heat treatment vs. four different extractions). One of the key mechanisms in developing volatile compounds is Maillard reaction, known as a non-enzymatic reaction [25]. Approximately 3500 volatile compounds are generated through Maillard reactions in the presence of amino acids, reducing sugars, and heat [25]. This additional heat largely governs the reaction by affecting the rate of volatile compound generation [25]. In general, low molecular weight decomposition products of amino acids and carbohydrates such as aldehydes and ketons are generated through Maillard reactions [25]. Based on the result of this study, 4 min roasting at 150 °C would be the optimum condition to retain the high amounts of volatile compounds in *P. frutescens* Britton.

#### 2.1.2. Sensory Description by GC-Olfactometry Installed in GC-MS

A sniffing test of identified volatile compounds was performed by GC-olfactometry in order to describe the sensory attributes of *P. frutescens* Britton during the roasting process. The result of sniffing test by three trained panelists is shown in Table 2. Interestingly, methyl benzoate described as perilla odor gave the highest olfactory intensity (4) throughout the whole roasting time. This result was consistent with our volatile compound results. Although the content of methyl benzoate showed an increase and decrease trend as explained in the previous section, the intensity seems strong enough to be perceived by the panelists. Another major volatile compound identified by GC-MS, limonene, described as minty odor was perceived till 4 min at the lowest intensity (1). This result was also in agreement with our GC-MS result for limonene which was detected up to 4 min with relatively lower concentration. Whereas in the GC-MS result, azulene was in a small quantity, the sensory attributes of azulene such as spicy, medicinal herb, and garlic odors were perceived intensely (4 at 0 and 2 min, 3 at 6 min). A previous study detected 13 volatile compounds in the leaves of *P. frutescens* Britton via a GC-olfactometry sniffing test and the most intensely perceived compounds were perilla ketone, (*Z*)-3-hexenal (green), and egoma ketone [24]. In the present study, α-terpinene and γ-terpinene which elicit bitter odors were perceived with strong intensities at 0, 4 and 3 min, respectively, but they were not perceived at all in the roasted samples. As the bitter odor in fresh vegetables is a major cause of deterring consumers from purchase, elimination of the bitter odors through roasting may act positively in increasing perilla consumption.

### 2.2. Constituent and Free Amino Acids Analyses and E-Tongue Analysis

#### 2.2.1. Constituent Amino Acids Analysis

The contents of constituent amino acids in *P. frutescens* Britton during the roasting were measured using an acid hydrolysis method and the results are shown in Table 3. 

Glutamic acid was the most abundant amino acid, followed by aspartic acid, alanine, glycine, leucine, and phenylalanine at 0 min. Overall, the contents of these amino acids showed various patterns without substantial changes. The contents of glutamic acid, aspartic acid, and leucine increased from 14.68 ± 0.46, 12.45 ± 0.52, and 8.01 ± 0.08 at 0 min to 15.30 ± 0.38, 12.65 ± 0.32, and 8.15 ± 0.11 at 8 min, respectively. Glutamic acid alone elicits a sour taste but when it reacts with sodium, it becomes a good flavor enhancer, known as umami taste [26,27,28]. Aspartic acid and leucine are known to have sour and bitter tastes, respectively [27]. The contents of alanine and glycine, on the other hand, decreased from 8.72 ± 0.22 and 8.03 ± 0.21 at 0 min to 8.64 ± 0.21 and 7.60 ± 0.19 at 8 min, respectively. Both alanine and glycine contribute to sweet taste [27]. Phenylalanine, another compound eliciting bitter taste like leucine [27], showed a slight increase upon roasting from 7.47 ± 0.96 at 0 min to 8.10 ± 0.73 at 8 min. Based on the result of this study, *P. frutescens* Britton is mainly composed of constituent amino acids that have umami, sweet, and bitter tastes and these are typical constituent amino acids and relevant tastes found in medicinal herbs such as *Cynanchi wilfordii* Radix [26].

#### 2.2.2. Free Amino Acids Analysis

The contents of free amino acids in *P. frutescens* Britton during the roasting were measured by an acid hydrolysis method and the results are shown in Table 4. Alanine was the most prevalent, followed by valine, hydroxylysine, and γ-aminobutyric acid (GABA). The contents of alanine and GABA exhibited a significant decrease during the roasting from 37.33 ± 2.79 and 13.61 ± 0.01 at 0 min to 22.73 ± 1.60 and 8.96 ± 0.47 at 8 min, respectively. Alanine induces sweet taste as previously mentioned [27] and the decreased alanine content with the roasting showed a similar pattern with that of constituent amino acids. GABA is an amino acid inhibitory neurotransmitter that regulates physiological functions and muscle movement in mammals, and a metabolite that controls stress and metabolic pathways in plants [29,30]. The contents of valine and hydroxylysine, on the contrary, were increased from 20.24 ± 0.92 and 18.13 ± 0.28 at 0 min to 28.59 ± 0.36 and 30.73 ± 1.11 at 8 min, respectively. Valine elicits bitter taste and acts as a synergistic factor that enhances umami taste [27]. Hydroxylysine induces sweet and bitter tastes [27]. Based on this result, *P. frutescens* Britton mainly consisted of free amino acids that induce sweet and bitter tastes.

#### 2.2.3. E-Tongue Analysis

**Figure 1:**
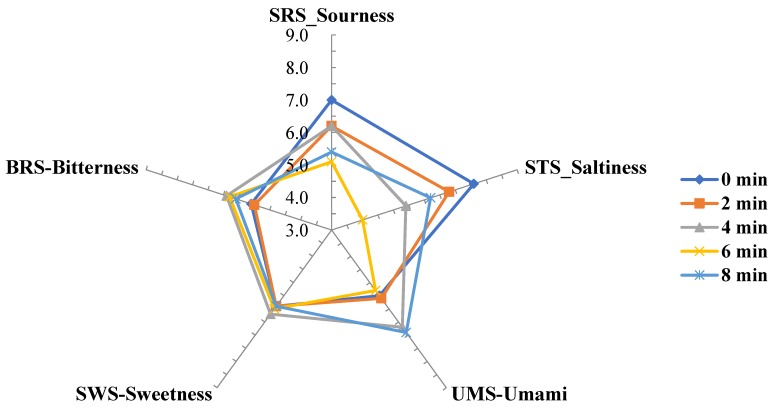
Chemical measures associated with sensory attributes of P. frutescens Britton var. acuta Kudo during the roasting using E-tongue.

E-tongue analysis was performed to investigate chemical measures associated with the sensory characteristics of *P. frutescens* Britton during the roasting process. The e-tongue analysis result is shown in Figure 1. The relative taste comparison of *P. frutescens* Britton during the roasting showed that umami and bitterness increased whereas sourness and saltiness decreased. Sweetness remained unchanged. These results can be explained in relation to the constituent and free amino acids compositions explained in the previous section of this study. Increase of umami taste may be associated with the increase of glutamic acid and that of bitterness would be related to the increase of leucine, phenylalanine, and valine as shown in Table 3 and Table 4. Although the amino acids eliciting sweet tastes including alanine and glycine increased, sweetness analyzed by e-tongue did not show a marked change over time. In case of sourness, aspartic acid inducing sour taste increased but corresponding sourness by e-tongue decreased. Such a discrepancy is probably because amino acids are not the sole factors of taste development but many other components including sugars, fatty acids, and volatile compounds participate complexely in taste development [27]. Recently, e-tongues have widely utilized to objectively measure tastes in many studies, such as investigation of the taste properties of fermented soybeans with mycelia of *Tricholoma matsutake* and *Bacillus* sp. [28], analysis of chemical compounds that may affect sensory properties of different parts of *Wasabi koreana* Nakai [30], discrimination of geographical origins of red ginseng [31], and investigation of the sensory properties of commercial seasonings [32].

### 2.3. Chemical Characteristics

#### 2.3.1. Total Phenolic Content (TPC)

Total phenolic content in *P. frutescens* Britton was determined by the Folin-Ciocalteu’s method and the results are shown in Table 5. Total phenolic content is an important indicator of antioxidant capacity [30]. The highest total phenolic content appeared at 4 min and 6 min (0.11 ± 0.01). Phenolic compounds are known to be thermally stable and phenolic antioxidants produced by roasting such as reductone would greatly attribute to the increase of total phenolic content [26,33]. The increased total phenolic content, however, decreased to 0.04 ± 0.01 at 8 min. In the study by Yun et al. [11] examining optimum roasting conditions of *P. frutescens* leaves for a tea, roasting at 160 °C for 25 min yielded the highest total phenolic content and the roasting beyond these conditions caused a decrease of the content, which is in agreement with the results of this study. It is therefore necessary to perform more studies investigating the optimum time and temperature to obtain high total phenolic content. Based on the results of this study, roasting at 150 °C for 6 min would be recommended to produce the highest total phenolic contents in *P. frutescens* Britton.

#### 2.3.2. Antioxidant Capacity

Antioxidant capacity was determined by measuring DPPH (1,1-diphenyl-2-picrylhydrazyl) and ABTS (2,2′-azino-bis-3-ethyl benzothiazoline–6-sulphonic acid) radical scavenging activities and the results are shown in Table 5. The results of both DPPH and ABTS analyses showed a similar pattern. IC_50_ values decreased with roasting at 2–6 min (84–90 mg) and (3 mg) in DPPH and ABTS analyses, respectively, indicating that *P. frutescens* Britton had paramount antioxidant capacity at 2–6 min roasting. Similar to the increase of total phenolic content, thermal processing seems to induce the production of antioxidants and an increase of free polyphenols that can act as electron donors [34]. The antioxidant capacity substantially decreased at 8 min, showing an identical pattern with the result of total phenolic content shown in this study. A recent study by Kim et al. [26], investigated the antioxidative properties of roasted *Cynanchi wilfordii* Radix and found that the sample roasted at 180 °C for 4 min produced the highest antioxidant capacity with no clear pattern with the increase of temperature and time. Both our study and the one by Kim et al. [26], indicate that roasting at high temperature for a longer time does not necessarily yield higher antioxidant capacity. Further investigation would be required for discovering the optimum roasting conditions of *P. frutescens* Britton in regards to antioxidant capacity.

#### 2.3.3. Browning Intensity

Absorbance at 420 nm was measured to determine browning intensity and the result is shown in Table 5. Browning intensity of *P. frutescens* Britton tended to increase with roasting time. The absorbance showed 0.15 ± 0.00 at 0 min and reached the highest at 6 min, 0.33 ± 0.00. It is generally accepted that increased brown color at high temperature over time is mainly caused by Maillard browning [25]. After roasting at 6 min, however, the intensity decreased to 0.23 ± 0.00. Another study also observed a similar result in that brown color developed by heat treatment in *Polygonatum odoratum* roots increased up to a certain time point and decreased afterward [35]. Kwon et al., explained that the substrates involved Maillard reaction such as reducing sugars and amino acids are depleted over time, thereby exerting a decrease in brown color development [35]. Considering that browning intensity is a critical determinant that can influence on consumer preference as well as food quality [25], investigation of proper roasting conditions is deemed necessary in respect to browning intensity.

## 3. Materials and Methods

### 3.1. Materials and Sample Treatment

*Perilla frutescens* Britton *var. acuta* Kudo leaves cultivated at Sancheong Herb Medicinal Cooperative (Sancheong, Republic of Korea) were purchased. The sample was prepared by the method outlined by Kim et al. [36]. The sample was air dried at 60 °C for moisture removal after which it was subjected to pulverization using a 50-mesh sieve. A sample (40 g) was roasted at 150 °C for 0, 2, 4, 6, and 8 min on a frying pan and an infrared thermometer (Infrared DT-8380, InnoCal Solutions Co., Vernon Hills, IL, USA) was used to measure the temperature. The roasted and pulverized sample was frozen and kept at −40 °C until further analyses.

### 3.2. Volatile Compound Identification and Sniffing Test Using Gas Chromatography-Olfactometry (GC-Olfactometry)

#### 3.2.1. SPME and GC-MS

Solid-phase microextraction (SPME) fiber coated with polydimethylsiloxane (PDMS) (Supelco, Bellefonte, PA, USA) was used to collect the volatile compounds. Pentadecane (10 μg) was dissolved in ethyl ether (1 mL) and utilized as an internal standard. The sample was heated in a 60 °C heating block for 20 min and the volatile compounds were collected for 30 min in the SPME fiber. After 10 min desorption, the collected volatile compounds were analyzed by gas chromatography-mass spectrometry (GC-MS, Agilent 7890A and 5975C, Agilent Technologies, Santa Clara, CA, USA). An HP-5MS column (30 m × 0.25 mm i.d. × 0.25 μm film thickness) was used for the analysis. Oven temperature was maintained at 40 °C for 5 min and then elevated to 200 °C at a rate of 5 °C/min. The injector temperature was set to 220 °C. Helium carrier gas flowed at 1.0 mL/min and the split ratio was 1:10. Compounds separated from the total ionization chromatogram (TIC) were identified using the mass spectrum library (NIST 12), ion fragmentation pattern, and a reference [37]. Volatile compounds were determined by semiquantative method based on converting into peak areas of the internal standard. The retention index (RI) was calculated using Equation (1) below:

RI*x* = 100*n* + 100 ((*tRx* − *tRn*)/ (*tRn*+1 − *tRn*)) (1)

where, RI*x* is the RI of the unknown compound, *tRx* is the retention time of the unknown compound, *tRn* is the retention time of the *n*-alkane, and *tRn*+1 is retention time of the next *n*-alkane. *tRx* is between *tRn* and *tRn*+1 (*n* = number of carbon atoms).

#### 3.2.2. Sniffing Test Using GC-Olfactometry Installed in GC-MS

A sniffing test of volatile compounds separated from GC/MS was carried out using an GC-olfactometry setup with a heated mixing chamber (ODP 3, Gerstel Co., Linthicum, MD, USA) mounted on the spectrometer. The conditions of GC-MS for the sniffing test were the same as described above, Section 3.2.1. Since olfactory recognition differs individually and olfactory sensitivity decreases over time, this study included three trained subjects in the sniffing test [38]. Recognized olfactory intensity was measured by a sensor with four intensity levels. Higher number indicates stronger olfactory intensity.

### 3.3. Electronic Tongue and Amino Acids Analyses

#### 3.3.1. Constituent Amino Acid Composition

To determine the contents of constituent amino acids, acid hydrolysis method using hydrochloric acid (HCl) was used. The sample (0.1 g) and 6 N HCl (3 mL) placed in a 20 mL flask were stirred for 10 min. The stirred sample was heated in a 110 °C preheated heating block (Thermo Fisher Scientific Co., Rockford, IL, USA) for 24 h, to induce the acid hydrolysis of proteins to amino acids. A portion (20 μL) of the mixture was taken and diluted with commercially available sodium dilution buffer (1 mL) for amino acid analyzer (Biochrom Ltd., Cambridge, UK). Then, 1 mL of this solution was filtered with a 0.2 μm membrane filter, followed by quantification of constituent amino acids using an automated amino acid analyzer (L-8900, Hitachi HighTech, Tokyo, Japan).

#### 3.3.2. Free Amino Acid Composition

To assess the contents of free amino acids, a sample (1 g) dissolved in methanol (20 mL) was stirred for 10 min and centrifuged at 3000 rpm for 20 min. The supernatant was then dissolved in 25 mL of the sample dilution buffer and after an addition of sulfosalicylic acid (20 mL), the sample was incubated at 4 °C for an hour. The sample was centrifuged at 3000 rpm for 20 min and then filtered with a 0.2 μm membrane filter. The free amino acids were quantified by an automated amino acid analyzer (L-8900, Hitachi High Tech) [26].

#### 3.3.3. E-tongue Analysis

An electronic tongue module (E-tongue, ASTREE II, Alpha M.O.S, Toulouse, France) with seven sensors was used to investigate the compounds associated with taste characteristics of *P. frutescens* Britton upon roasting. Two sensors, SPS (spiciness) and GPS (metallic taste) sensors, were set as a reference. SRS (sourness), STS (saltiness), UMS (umami), SWS (sweetness) and BRS (bitterness) sensors represented for 5 basic tastes. The sample, 1 g, was extracted with 100 mL distilled water at 60 °C for 10 min and filtered with Whatman No. 1 filter paper (GE Healthcare, Little Chalfont, UK). The analysis was performed seven times and taste patterns were analyzed by multivariate analysis. The responses from the sensor were converted to scores ranged 1 to 12. Descriptive analysis was given for the resulted taste distribution and the results were shown in a radar plot, which indicates relative comparison among the tastes [39].

### 3.4. Chemical Characteristics

#### 3.4.1. Total Phenolic Content (TPC)

Total phenolic content of the sample was measured by Folin-Ciocalteu’s method [40]. The sample (1 mg/mL) was diluted with distilled water. Forty μL of the solution was mixed with distilled water (200 μL) and then 2 N Folin-Ciocalteu’s reagent (200 μL, Sigma-Aldrich Co., St. Louis, MO, USA) was added and mixed thoroughly for 30 s. Subsequently, 30% Na_2_CO_3_ (600 μL, Sigma-Aldrich Co.) and distilled water (160 μL) were added to the solution and incubated at room temperature for 2 h. Total phenolic contents of the sample were determined by absorbance measurement at 750 nm. Gallic acid (0–500 μg/mL) was used as a standard and treated the same procedure as for the sample to quantify total phenolic compounds. Total phenolic content was calculated from the calibration curve.

#### 3.4.2. Antioxidant Capacity

To investigate antioxidant capacity in *P. frutescens* Britton, DPPH (1,1-diphenyl-2-picrylhydrazyl) and ABTS (2,2′-azino-bis-3-ethylbenzothiazoline–6-sulphonic acid) radical scavenging activities were measured. DPPH radical scavenging activity was measured using a slight modification of a method described by Blois [41]. The sample was diluted with distilled water (0.1, 1, and 10 mg/mL). Next, 0.1 mM DPPH (1 mL, dissolved in 99% ethanol, Sigma-Aldrich Co.) was added to 10 μL of diluted sample. The mixture was incubated in the dark at 37 °C for 30 min. Absorbance was taken at 517 nm and radical scavenging activity was determined by the equation shown below:

Radical scavenging activity (%) = (1 − absorbance of sample/absorbance of control) × 100(2)

Based on the radical scavenging activity, the IC_50_ value (the sample concentration where radical scavenging activity reaches 50%) was calculated.

ABTS (2,2′-azino-bis-3-ethyl benzo thiazoline–6-sulphonic acid) radical scavenging activity was measured by adapting the method by Van den Berg et al. [42]. 14 mM ABTS solution and 4.9 mM potassium persulfate solution were mixed in a 1:1 ratio and reacted in the dark for 24 h. The mixture was diluted with PBS buffer to make absorbance 0.70 ± 0.02 at 734 nm, after which it was used as a working solution. The sample was diluted with distilled water (0.1, 1, and 10 mg/mL), and 20 µL of each diluted sample was taken and mixed with 180 μL of the working solution. The mixture was subsequently incubated in the dark at 37 °C for 10 min and absorbance was measured at 734 nm. Radical scavenging activity was calculated using the same formula above and IC_50_ value was calculated accordingly.

#### 3.4.3. Browning Intensity

To determine browning intensity of *P. frutescens* Britton upon roasting, the sample (1 g) was extracted with 100 mL of distilled water at 60 °C for 10 min. Extracted solution (200 µL) was placed in a 96-well plate and absorbance was measured at 420 nm using a spectrophotometer (Multiskan Go, Thermo-Fisher Scientific Co., Vantaa, Finland). Higher absorbance indicates higher browning intensity.

## 4. Conclusions

For extending the application of *P. frutescens* Britton as a food and food ingredient, this study investigated volatile compounds, sniffing test-assisted sensory properties, constituent and free amino acid compositions, taste description by e-tongue, and chemical characteristics, including TPC, antioxidative capacity, and browning intensity in *P. frutescens* Britton after roasting at 150 °C for various time periods. Of 142 volatile compounds identified, methyl benzoate and limonene were the most abundant, and these were also detected as major compounds in the sniffing test-assisted sensory description. Amino acid analyses resulted that among the constituent amino acids, the contents of glutamic acid, aspartic acid, and leucine, which induce umami taste, sour taste, and bitter taste, respectively, increased over the roasting time. Among free amino acids, the contents of valine and hydroxylysine eliciting bitter and bitter and sweet tastes, respectively, increased with roasting. The changes of amino acid composition by roasting were only somewhat matched to the taste description by e-tongue. The changes in sweetness and sourness analyzed by e-tongue did not correspond to the result of amino acid composition. For the chemical properties, TPC, antioxidative capacity, and browning intensity showed an increase pattern with roasting but decreased back after a certain time point (8 min). The result of this work provides baseline data on volatile and chemical compounds that may affect sensory properties and chemical properties of *P. frutescens* Britton after different roasting times, increasing the feasibility of perilla utilization as a food and food ingredient in both the food industry and the cooking environment.

## Figures and Tables

**Table 1:** Changes in volatile compounds in *P. frutescens* Britton *var. acuta* Kudo during the roasting.

Compounds ^1)^	Retention Time (min)	Retention Index	Roasting Time (min)				
			0	2	4	6	8
			Relative Concentration (μg/100 g)				
**Alcohols (16)**							
Isooctanol	17.71	1088	0.07	ND ^2)^	ND	ND	ND
3,7-Dimethyl-1,6-octadien-3-ol	18.29	1102	ND	2.70	ND	ND	ND
Linalool	18.31	1103	1.64	ND	4.15	3.52	0.50
2-Ethyl-1-decanol	18.41	1107	0.46	ND	ND	ND	ND
3,4-Dimethyl cyclohexanol	18.62	1114	ND	0.36	ND	ND	ND
3,5-Dimethyl cyclohexanol	18.64	1115	0.29	ND	0.31	ND	0.26
2-Ethyl-1-dodecanol	18.73	1118	0.20	ND	ND	ND	ND
3,7,11-Trimethyl-1-dodecanol	18.83	1122	0.13	ND	ND	ND	ND
8-Methylene-2-exo-noradamantanol	21.81	1226	ND	ND	1.35	ND	ND
2-Methylene cycloheptanol	22.25	1242	ND	ND	ND	0.30	ND
1-Tridecanol	22.32	1245	ND	ND	ND	ND	0.12
4-1-Methyl ethenyl-1-cyclohexene-1-methanol	24.02	1306	ND	1.48	ND	ND	ND
Perilla alcohol	24.02	1306	ND	ND	1.93	ND	ND
1-Eicosanol	27.62	1464	ND	0.66	ND	ND	ND
Isopulegol	28.34	1501	ND	ND	ND	0.22	ND
1-Ethynyl-2-methyl cyclohexanol	29.35	1544	0.34	ND	ND	ND	ND
**Aldehydes (5)**							
6-Oxononanal 2-Undecenal Citral	18.64 21.98 23.19	1232 1115 1277	ND 0.31 ND	ND ND ND	ND ND 0.17	0.30 ND 0.36	ND ND ND
2-Eehoxybenzaldehyde	23.60	1291	ND	ND	ND	ND	0.48
Caryophyll-5-en-12-al	30.58	1596	ND	0.65	ND	ND	ND
**Esters (2)**							
Phosphonic acid, dioctadecyl ester	26.20	1393	ND	0.28	ND	0.21	ND
2-Ethyl hexyl ester	29.00	1529	1.42	0.92	ND	ND	0.78
**Hydrocarbons (110)**							
5-Methyl styrene-1,6-heptadien-3-yne	11.74	921	0.13	ND	ND	ND	ND
1,5-Cyclooctadiyne	11.72	921	ND	0.24	ND	ND	ND
δ-3-Carene	12.87	956	ND	ND	0.10	ND	0.10
α-Phellandrene	12.87	956	ND	ND	ND	0.06	ND
2,6,6-Trimethyl bicyclo hept-2-ene	13.06	962	ND	3.26	ND	ND	ND
α-Pinene	13.08	963	2.65	ND	6.42	4.26	0.10
3,7-Dimethyl-1,3,6-octatriene	13.10	963	ND	ND	ND	ND	0.76
6-Methylene bicyclo-3,2,0-heptane	13.35	970	ND	ND	ND	0.13	ND
4-Methyl bicyclo-3,2,1-octan-3-ene	13.36	971	ND	ND	0.11	ND	ND
Sabinene	14.38	998	ND	ND	2.05	0.62	0.56
2-β-Pinene	14.50	1002	3.02	3.91	5.65	3.59	ND
β-Myrcene	14.90	1014	ND	0.44	1.31	0.57	ND
Cyclofenchene	14.93	1014	0.32	ND	0.85	ND	ND
2,6,6-Trimethylbicyclo-3,1,1-hept-2-ene	14.93	1014	ND	ND	ND	ND	0.17
α-Fenchene	15.35	1026	ND	ND	ND	0.54	ND
6,6-Dimethyl-2-methylene bicyclo-3,1,1-heptane	15.35	1026	0.45	ND	ND	ND	ND
1-Methyl-5-1-methyl ethenyl cyclohexene	15.36	1027	ND	ND	ND	ND	0.09
2,4-Dimethyl-1-decene	15.93	1042	ND	ND	ND	ND	0.04
4-Ethyl-1,2-dimethyl benzene	16.00	1044	ND	0.13	ND	ND	ND
Limonene	16.15	1048	54.66	58.18	137.24	60.92	12.56
2,6,7-Trimethyl decane	16.85	1066	ND	0.17	ND	ND	ND
2,6-dimethyl octane	16.88	1067	ND	ND	ND	0.35	0.29
1-ethenyloxy octadecane	16.95	1069	ND	0.14	ND	ND	ND
2,4-dimethyl heptane	16.97	1069	ND	ND	ND	0.12	0.35
2,4-Dimethyl hexane	16.97	1069	ND	ND	ND	ND	0.08
β-Phellandrene	17.06	1072	ND	ND	0.18	ND	ND
l-Phellandrene	17.07	1072	ND	ND	ND	0.16	ND
γ-Terpinene	17.07	1072	0.14	0.17	ND	ND	ND
3,5-Dimethyl undecane	17.13	1073	ND	0.24	ND	0.24	0.13
2-Methyl decane	17.16	1073	ND	ND	ND	ND	0.15
2,8-Dimethyl undecane	17.52	1083	ND	0.57	0.38	ND	ND
11-1-Ethylpropyl heneicosane	17.54	1083	ND	ND	ND	0.42	ND
3,7-Dimethyl decane	17.54	1084	ND	ND	ND	ND	0.27
α-Terpinolene	17.97	1094	ND	0.78	1.02	0.67	ND
α-Terpinene	17.98	1095	0.63	ND	0.88	ND	ND
Undecane	18.23	1100	0.37	ND	ND	ND	0.35
5-(1-Methyl propyl)-Nonane	18.72	1118	ND	ND	0.18	ND	ND
Dodecane	18.74	1200	ND	ND	ND	ND	0.14
1,1′-Oxybis decane	18.83	1122	ND	0.15	0.14	0.06	ND
Dodecyloxy methyl oxirane	18.94	1126	ND	0.27	ND	ND	ND
3-Methyl tridecane	18.96	1127	0.20	ND	ND	ND	ND
2,3,6,7-Tetramethyl octane	19.03	1129	ND	0.16	ND	ND	ND
1-Fluoro dodecane	19.04	1130	ND	ND	ND	ND	0.12
3,7-Dimethyl nonane	19.04	1130	0.14	ND	ND	0.15	ND
3-Ethyl-3-methyl heptane	19.20	1135	ND	ND	0.09	ND	ND
1,1,1,2-Tetrafluoro-2-tridecene	19.21	1136	ND	ND	ND	ND	0.12
4-Methyl undecane	19.96	1162	ND	0.37	ND	0.34	ND
3-Methyl decane	19.97	1162	ND	ND	0.32	0.18	ND
1-Chloro hexadecane	19.97	1162	ND	ND	ND	ND	0.17
2,6,10-Trimethyl dodecane	19.97	1162	0.28	ND	ND	ND	ND
2-Methyl undecane	20.10	1166	0.16	ND	ND	ND	ND
3-Ethyl octane	20.14	1168	ND	0.53	ND	ND	ND
2,6,10,14-Tetramethyl hexadecane	20.15	1168	ND	ND	0.25	ND	ND
2,6,11-Trimethyl dodecane	20.16	1168	0.25	ND	0.24	ND	ND
3,6-Dimethyl undecane	20.16	1168	ND	ND	ND	ND	0.19
3,5,24-Trimethyl tetracontane	20.28	1172	ND	ND	ND	0.53	ND
1-Methoxymethoxy dodecane	20.65	1185	0.17	ND	ND	ND	ND
1,1,4,4,7,7,-Hexamethyl cyclononane	20.73	1187	0.13	ND	ND	ND	ND
2,3,5,8-Tetramethyl decane	20.74	1187	ND	ND	ND	0.19	ND
Azulene	20.86	1192	1.41	1.80	1.45	1.33	ND
Naphthalene	20.87	1192	ND	ND	ND	ND	0.84
Camphene	21.02	1197	ND	0.53	0.17	0.10	ND
1-Methyl-4-1-methyl ethylidene cyclohexene	21.03	1197	ND	ND	ND	0.62	ND
Dodecane	21.12	1200	1.97	2.41	2.37	2.44	1.79
7-Butyl bicyclo-4,1,0-heptane	21.26	1205	ND	ND	ND	0.25	ND
Pulegone	21.35	1209	ND	0.62	ND	ND	ND
Decahydro naphthoxirene	21.36	1209	0.40	ND	ND	ND	ND
2,5-Dimethyl undecane	21.49	1214	ND	0.47	ND	ND	ND
2,6-Dimethyl undecane	21.51	1215	0.37	ND	0.39	0.42	0.15
4,6-Dimethyl undecane	21.51	1215	ND	ND	ND	ND	0.27
*cis*-Ocimene	21.81	1226	ND	0.94	ND	ND	ND
1,3-Dimethyl butyl cyclohexane	22.25	1242	ND	ND	ND	ND	0.16
Methoxy methoxy cyclooctane	22.56	1254	ND	ND	ND	0.28	ND
2-2-Tetrafuryl methyl tetrahy dropyran	22.73	1260	ND	0.43	ND	ND	ND
4,4-Dipropylheptane	22.74	1260	0.30	ND	ND	ND	ND
2,3,6-Trimethyl decane	22.86	1265	ND	0.60	ND	ND	ND
2,3-Dimethyl undecane	22.87	1265	0.49	ND	ND	ND	ND
2,3,5-Trimethyl decane	22.87	1265	ND	ND	0.44	ND	ND
3-Methyl undecane	22.87	1265	ND	ND	ND	0.51	ND
3-Methyl dodecane	23.07	1272	ND	ND	0.32	ND	ND
2,6-Dimethyl heptadecane	23.11	1273	0.73	ND	ND	ND	0.23
3-Methyl nonane	23.11	1274	ND	ND	ND	0.67	ND
2,5,6-Trimethyl-1,3,6-heptatriene	23.25	1279	1.78	ND	ND	ND	ND
Tricyclodecane	23.26	1279	ND	3.46	ND	ND	ND
Tridecane	23.83	1300	4.50	5.36	5.09	5.15	3.33
2-Methyl-1,3-cyclononadiene	24.81	1338	ND	0.52	ND	ND	ND
6-Isopropylidene-1-methyl Bicyclo-3,1,0-hexane	24.82	1339	ND	ND	0.81	ND	ND
4,4-Dipropyl heptane	25.21	1354	ND	0.38	ND	ND	0.09
5-Methyl tetradecane	25.21	1355	0.28	ND	ND	ND	ND
2,7-Dimethyl undecane	25.22	1355	ND	ND	ND	ND	0.19
3-Methyl tridecane	25.64	1371	ND	0.58	ND	ND	ND
α-Cubebene	26.01	1386	0.73	1.13	ND	0.98	ND
Copaene	26.01	1386	ND	ND	1.12	ND	0.41
Methyl cyclooctane	26.19	1392	0.27	ND	ND	ND	ND
β-Bourbonene	26.27	1395	ND	ND	0.56	0.35	ND
Tetradecane	26.39	1400	2.81	3.12	3.15	2.90	1.84
Decyl oxirane	26.67	1415	ND	ND	ND	ND	0.27
α-Longipinene	27.01	1433	ND	ND	ND	0.36	ND
Caryophyllene	27.16	1441	ND	24.84	52.34	32.33	5.49
trans-Caryophyllene	27.16	1441	26.04	ND	ND	ND	ND
Sinularene	27.35	1451	ND	1.13	ND	ND	ND
Aromadendrene	27.36	1451	ND	ND	ND	1.05	ND
1-Hexyl-1-nitrocyclohexane	27.63	1465	ND	ND	ND	ND	0.30
α-Humulene	27.99	1483	1.97	ND	ND	ND	0.45
α-Caryophyllene	28.00	1483	ND	ND	ND	1.99	ND
Germacrene D	28.65	1514	1.36	3.78	5.15	1.73	ND
α-Farnesene	28.76	1519	5.02	ND	1.72	0.59	ND
3,7,11-Trimethyl-1,3,6,10-dodecatetraene	28.77	1519	ND	ND	ND	6.88	ND
Bicyclogermacrene	29.01	1530	ND	ND	ND	1.35	ND
Dispiro-4,2,4,2-tetradecane	29.23	1539	ND	ND	ND	0.35	ND
**Ketons (1)**							
5,6-Methylidene-2-norbornen-7-one	11.71	921	ND	ND	0.24	ND	ND
**Heterocyclic (3)**							
4-Ethyl-2,6-dimethyl pyridine	29.55	1045	0.56	ND	ND	ND	ND
3-Methyl-2,3-dihydro benzofuran	16.02	1553	ND	ND	ND	0.09	ND
4-Ethyl-2,6-dimethyl pyridine	30.70	1601	ND	0.60	ND	ND	ND
**Miscellaneous (5)**							
α-Terpinenyl acetate	21.03	1118	0.45	ND	ND	ND	ND
Dihexyl sulfide	18.72	1197	ND	ND	ND	0.26	ND
*O*-Decyl hydroxylamine	22.74	1260	ND	ND	ND	ND	0.21
Methyl benzoate	23.39	1284	88.08	112.46	203.10	122.67	21.31
Calarene epoxide	28.22	1494	ND	0.27	ND	ND	ND

^1)^ Compounds were tentative identified by GC/MSD library connecting HP5-MS column and their retention indices(RIs). ^2)^ NS corresponds not detected.

**Table 2:** Sensory description and odor intensity of *P. frutescens* Britton *var. acuta* Kudo during the roasting using GC-olfactometry.

Major Volatile Compounds ^1)^	Odor Description	Odor Intensity				
		Roasting Time (min)				
		0	2	4	6	8
Limonene	Mint	1	1	1	-	-
α-Terpinene	Bitter	4	-	-	-	-
Linalool	Sweet	1	-	-	2	1
Azulene	Spicy, Medicinal herb, Garlic	4	4	-	3	-
2,6-Dimethylundecane	Oil	2	-	-	2	-
Methyl benzoate	Perilla	4	4	4	4	4
γ-Terpinene	Bitter	3	-	-	-	-
3,7-Dimethyl-1,6-octadien-3-ol	Sweet	2	-	-	-	-
2,5-Dimethylundecane	Oil	-	3	-	-	-
β-Phellandrene	Spicy, Grass	-	-	2	2	-
α-Terpinolene	Spicy, Green onion	-	3	1	1	-
α-Pinene	Spicy, Pepper	-	-	-	-	2
4,4-Dipropylheptane	Roasted	-	-	-	-	1

^1)^ Compounds were tentative identified by GC/MSD library connecting HP5-MS column and their retention indices(RIs).

**Table 3:** Constituent amino acids in *P. frutescens* Britton *var. acuta* Kudo during the roasting.

Constituent Amino Acid (% Perilla)	Roasting Time (min)					Inc. Dec. Ratio ^2)^ (% Perilla)
	0	2	4	6	8	
Aspartic acid	12.45 ± 0.52 ^a^^,1)^	11.99 ± 0.13 ^a^	12.43 ± 0.04 ^a^	12.00 ± 0.13 ^a^	12.65 ± 0.32 ^a^	1.01
Threonine	5.27 ± 0.20 ^a^	5.03 ± 0.06 ^ab^	4.90 ± 0.02 ^b^	5.04 ± 0.05 ^ab^	5.17 ± 0.13 ^ab^	0.98
Serine	6.91 ± 0.24 ^ab^	6.49 ± 0.07 ^b^	6.68 ± 0.01 ^ab^	6.51 ± 0.07 ^b^	7.05 ± 0.18 ^a^	1.02
Glutamic acid	14.68 ± 0.46 ^ab^	14.33 ± 0.13 ^a^	14.60 ± 0.02 ^ab^	14.35 ± 0.14 ^b^	15.30 ± 0.38 ^a^	1.04
Glycine	8.03 ± 0.21 ^a^	7.40 ± 0.12 ^b^	7.49 ± 0.03 ^b^	7.18 ± 0.06 ^b^	7.60 ± 0.19 ^b^	0.94
Alanine	8.72 ± 0.22 ^a^	8.31 ± 0.12 ^ab^	8.49 ± 0.04 ^ab^	8.12 ± 0.08 ^b^	8.64 ± 0.21 ^a^	0.99
Cysteine	3.23 ± 0.16 ^a^	2.57 ± 0.10 ^b^	3.19 ± 0.04 ^a^	2.73 ± 0.01 ^b^	3.44 ± 0.08 ^a^	1.06
Valine	4.21 ± 0.13 ^ab^	3.93 ± 0.05 ^c^	3.95 ± 0.01 ^c^	4.03 ± 0.04 ^bc^	4.29 ± 0.12 ^a^	1.02
Methionine	2.44 ± 0.12 ^a^	1.79 ± 0.34 ^b^	1.21 ± 0.03 ^c^	1.56 ± 0.02 ^bc^	1.14 ± 0.02 ^c^	0.47
Isoleucine	1.84 ± 0.02 ^bc^	2.02 ± 0.01 ^a^	1.80 ± 0.01 ^c^	2.03 ± 0.01 ^a^	1.90 ± 0.03 ^b^	1.03
Leucine	8.01 ± 0.08 ^b^	8.65 ± 0.07 ^a^	8.04 ± 0.02 ^b^	8.47 ± 0.05 ^a^	8.15 ± 0.11 ^b^	1.02
Tyrosine	3.95 ± 0.28 ^b^	4.28 ± 0.02 ^ab^	4.33 ± 0.03 ^a^	4.32 ± 0.03 ^a^	4.15 ± 0.12 ^ab^	1.05
Phenylalanine	7.47 ± 0.96 ^b^	8.52 ± 0.10 ^ab^	9.02 ± 0.04 ^a^	8.60 ± 0.05 ^ab^	8.10 ± 0.73 ^ab^	1.08
Lysine	5.32 ± 0.62 ^ab^	6.03 ± 0.01 ^a^	5.99 ± 0.04 ^ab^	6.00 ± 0.04 ^a^	4.96 ± 0.57 ^b^	0.93
Histidine	2.41 ± 0.26 ^b^	3.00 ± 0.19 ^a^	2.92 ± 0.06 ^ab^	3.03 ± 0.08 ^a^	2.43 ± 0.35 ^b^	1.01
Arginine	5.05 ± 0.08 ^a^	5.65 ± 0.76 ^a^	4.97 ± 0.01 ^a^	6.02 ± 0.67 ^a^	5.04 ± 0.12 ^a^	0.99

Data represent the mean ± SD in triplicate; ^1)^ Means with different letters (a–e) within a row are significantly different by Tukey’s multiple range test (*p* < 0.05); ^2)^ Increase and decrease ratio was calculated based on amino acid contents at 0 and 8 min. The baseline ratio is 1.00 at 0 min.

**Table 4:** Free amino acids in *P. frutescens* Britton *var. acuta* Kudo during the roasting.

Free Amino Acid (% Perilla)	Roasting Time (min)					Inc. Dec. Ratio ^2)^
	0	2	4	6	8	(% Perilla)
Serine	1.76 ± 0.17 ^b,1)^	1.79 ± 0.14 ^b^	2.55 ± 0.09 ^a^	2.00 ± 0.18 ^b^	ND ^c^	0
Glycine	7.42 ± 0.59 ^a^	7.82 ± 0.30 ^a^	8.46 ± 0.24 ^a^	8.11 ± 0.76 ^a^	8.97 ± 1.03 ^a^	1.07
Alanine	37.33 ± 2.79 ^a^	29.90 ± 5.99 ^a,b^	28.96 ± 0.66 ^b^	26.98 ± 1.66 ^b^	22.73 ± 1.60 ^b^	0.54
Valine	20.24 ± 0.92 ^b^	22.76 ± 2.53 ^a,b^	21.79 ± 0.55 ^b^	24.54 ± 1.51 ^ab^	28.59 ± 0.36 ^b^	1.25
Isoleucine	1.51 ± 0.06	ND ^3)^	ND	ND	ND	0
GABA	13.61 ± 0.01 ^a,b^	15.00 ± 0.11 ^a^	13.86 ± 0.15 ^b^	12.68 ± 0.17 ^c^	8.96 ± 0.47 ^d^	0.58
Hydroxylysine	18.13 ± 0.28 ^c^	22.72 ± 2.19 ^bc^	24.38 ± 0.35 ^b^	25.67 ± 0.46 ^ab^	30.73 ± 1.11 ^a^	1.50

Data represent the mean ± SD in triplicate; ^1)^ Means with different letters (a–d) within a row are significantly different by Tukey’s multiple range test (*p* < 0.05); ^2)^ Increase and decrease ratio was calculated based on amino acid contents at 0 and 8 min. The baseline ratio is 1.00 at 0 min. ^3)^ NS corresponds not detected.

**Table 5:** Total phenolic content, antioxidant activity, and browning intensity in *P. frutescens* Britton *var. acuta* Kudo.

	Roasting Time (min)				
	0	2	4	6	8
TPC (mg/mL) ^1)^	0.04 ± 0.01 ^d,^^2)^	0.11 ± 0.01 ^a^	0.08 ± 0.01 ^b^	0.11 ± 0.01 ^a^	0.04 ± 0.01 ^c^
DPPH(IC_50_) (mg)	123	84	86	90	138
ABTS(IC_50_) (mg)	4	3	3	3	7
Browning intensity (420 nm)	0.15 ± 0.01 ^e^	0.29 ± 0.01 ^b^	0.25 ± 0.01 ^c^	0.33 ± 0.01 ^a^	0.23 ± 0.01 ^d^

Data represent the mean ± SD in triplicate; ^1)^ TPC: total polyphenol content; ^2)^ Means with different letters (a–e) within a row are significantly different by Tukey’s multiple range test (*p* < 0.05).
